# Management of Chemotherapy Induced Cardiomyopathy

**DOI:** 10.2174/157340311799960681

**Published:** 2011-11

**Authors:** Abdulfattah Saidi, Rami Alharethi

**Affiliations:** Division of Cardiology, University of Utah Health Sciences Center, Salt Lake City, Utah 84132, USA

**Keywords:** Cardiomyopathy, Chemotherapy, Management.

## Abstract

Chemotherapy related cardiac dysfunction (CRCD) is a serious complication of anticancer therapy. CRCD can be classified into two types. Type I CRCD is exemplified by anthracyline- induced cardiac dysfunction and type II CRCD is exemplified by trastuzumab- induced cardiac dysfunction. The mechanism of cardiac toxicity in both types is not well defined. Certain risk factors may play a role in developing the cardiac injury, most importantly, the cumulative dose when dealing with anthracycline induced cardiotoxicity. Establishing an early diagnosis and initiating early treatment may be an important step in preventing irreversible cardiac injury especially in type I CRCD. Currently there are no guidelines developed specifically for the treatment of chemotherapy induced cardiomyopathy (CIC), however a few small studies support the use of neurohormonal antagonists in the treatment and prevention of CIC. Large multi- centers trials are needed to establish guidelines for CIC. Until then, we advocate following the American College of Cardiology/ American Heart Association (ACC/AHA) and Heart Failure Society of America (HFSA) guidelines. Additionally, a close collaboration between the patient’s cardiologist and oncologist is strongly recommended in order to establish a long term plan for the patient.

## INTRODUCTION

There has been tremendous progress in cancer therapy in recent years rendering many cases as treatable and curable. One potentially negative consequence of this progress is that a growing number of survivors are at a higher risk for cardiovascular disease due to the cardiotoxic nature of some of the therapeutic agents (1). Therefore, the close collaboration between oncologists and cardiologists is very important in the care of a cancer patient who is receiving a cardiotoxic agent as part of their treatment regimen (2). The major cardiac complications associated with chemotherapy are arrhythmia, pericarditis, myocardial ischemia and cardiomyopathy. Although a wide range of chemotherapy agents can cause cardiotoxicity (Table **1**), anthracyclines are the most common perpetrators. With the recent introduction of trastuzumab for the treatment of positive HER2 breast cancer there have been more reports of chemotherapy-induced cardiotoxicity. 

## DEFINITION

The Cardiac Review and Evaluation Committee (CRCE), has established criteria for the diagnosis of chemotherapy related cardiac dysfunction (CRCD) as: 1) cardiomyopathy characterized by a decrease in cardiac left ventricular ejection fraction (LVEF), either global or more severe in the septum; 2) symptoms of heart failure (HF) ; 3) associated signs of HF, including but not limited to S3 gallop, tachycardia, or both; and 4) decline in LVEF of at least 5% to less than 55% with accompanying signs or symptoms of HF, or a decline in LVEF of at least 10% to below 55% without accompanying signs or symptoms. The presence of any one of the four criteria is sufficient to confirm a diagnosis of CRCD (2).

## PATHOGENESIS

(CRCD) can be sub-classified into two types. Type I CRCD is exemplified by an anthracycline-associated cardiac dysfunction, with its underlying mechanism not yet well understood. The myocyte damage might be attributed to the production of oxygen free radicals and subsequent increase in oxidative stress (3). Iron hemostasis might also play a role in the myocardial injury as anthracyclines impair the iron metabolism pathways and cause iron accumulation in the cardiomyocytes (4). The cumulative dose of anthracycline (5) is strongly associated with cardiotoxicity, however, varying nature of responses to different doses of anthracyclines have been observed (6). Also the administration schedule, concomitant use of other cardiotoxic therapies, age, and female gender are important contributors towards the onset and progression of cardiomyopathy (7).

Type II CRCD on the other hand can be exemplified by a trastuzumab induced cardiomyopathy. Trastuzumab is a humanized monoclonal antibody approved by the FDA for the treatment of positive HER2 breast cancer. The mechanism of trastuzumab related cardiomyopathy is not well defined but the epidermal growth signal pathway (HER2) in the heart is implicated which suggests that trastuzumab cardiotoxicity is related to HER2 blockade (8, 9). In contrast to anthracycline induced cardiotoxicity, trastuzumab induced cardiac injury is not related to the cumulative dose. It is often reversible after treatment discontinuation and can be tolerated once again, if indicated, after recovery (10). The risk of developing trastuzumab cardiotoxicity increases in patients who receive concurrent anthracycline therapy especially if the cumulative doxorubicin dose is > 300 mg/m². Other risk factors include age of greater than 50 years, preexisting cardiac dysfunction, and high body mass index. Trastuzumab induced cardiotoxicity is usually asymptomatic and often presents with a decrease in LVEF and less often by overt heart failure (10).

## SCREENING AND DIAGNOSIS

LVEF should be assessed in cancer patients undergoing chemotherapy although there is no consensus as to the frequency and the mode. Exercise or dobutamine stress echocardiography may be a sensitive tool in the early detection of subclinical cardiomyopathy, providing an opportunity for therapeutic intervention before the development of overt left ventricle dysfunction (5). A baseline evaluation of LVEF needs to be obtained for comparison. Radionuclide gated ventriculogram (MUGA) or echocardiogram has been used in monitoring and diagnosing cardiac dysfunction. Cardiac biomarkers such as troponin I and natriuretic peptides have been studied with promising results (11, 12). Although endomyocardial biopsy is the gold standard for the diagnosis of type I chemotherapy induced cardiomyopathy, the data is still lacking with regards to type II. Unfortunately the invasive nature of this procedure limits its use.

## PREVENTION AND TREATMENT

In addition to decreasing the cumulative dose of anthracyclines, there are other approaches that may reduce the risk of developing type I CRCD. The administration of anthracyclines as infusions rather than as boluses, (13,14, 15) the structural modification of doxorubicin (16) and the liposomal encapsulation of doxorubicin (17) are all measures which may help in reducing the degree of cardiac toxicity. Dexrazoxane, an EDTA like chelator, may reduce the risk of cardiotoxicity when given with doxorubicin or epirubicin (16). However, its use has been limited to patients who receive a cumulative dose of doxorubicin ≥300 mg/m2 due to the potential impact on the antitumor efficacy (18)

Carvedilol, a β-blocker with antioxidant properties (19), might reduce the risk of anthracyclines induced cardiomyopathy. Kalay *et al* randomized 50 patients receiving anthracycline therapy to either carvedilol 12.5 mg once daily or placebo. There was no change in the LVEF in the carvedilol group after 6 months, on the other hand the LVEF significantly decreased by 17 percent in the placebo group. Given the small size of this study additional larger studies are needed (20).

Early animal studies demonstrated that the renin-angiotensin pathway plays an important role in type I CRCD (21-27). An open label single center clinical study by Cardinale *et al* randomized 114 high risk patients with elevated troponin I after receiving high dose anthracyclines, to receive either enalapril at a starting dose of 2.5 mg daily or placebo for one year (28). Twenty five patients (43%) in the control group had a decrease in the LVEF compared to none in the enalapril group (max dose was 16 ± 6mg). Additionally there was a significant increase in cardiac events in the control group mainly due to heart failure when compared to the enalapril group. As impressive as these results may be, they must be confirmed by a multicenter trial. Another study evaluated the use of an angiotensin II receptor blocker (ARB) to prevent acute cardiac injury in patients receiving standard Cyclophosphamide, Doxorubicin, Vincristine and Predinsolone (CHOP) (29). Forty patients with untreated non-Hodgkin’s lymphoma were randomized to receiving either valsartan 80 mg daily or placebo at the time of starting CHOP treatment. On day 3 after the initiating of the CHOP treatment the valsartan group had lower brain natriuretic peptide (BNP) levels, similar atrial natriuretic peptide levels, fewer changes in left ventricular end diastolic dimensions and QTc dispersions when compared to the placebo group. These interesting results although promising, are hampered by the very small sample size.

Cadeddu *et al*. examined the possible role of telmisartan (ARB) in preventing epirubicin induced cardiotoxicity. 49 patients were enrolled and randomized to receive either telmisartan or placebo. Patients in the telmisartan group had lower serum levels of pro-inflammatory cytokines (IL-6 and TNF-α) and reactive oxygen species when compared to the placebo group. Furthermore, at high doses of epirubicin there was a significant recovery of peak strain rate in the telmisartan group in comparison to the placebo group. This study concluded that telmisartan could reverse early myocardial impairment (30). 

A prospective, parallel group, randomized, controlled study that included one hundred and twenty five patients was conducted by Georgakopoulos *et al*. This study examined the doxorubicin induced clinical or subclinical cardiotoxicity in lymphoma patients after concomitant prophylactic therapy with metoprolol or enalapril. Though the differences were not statistically significant (likely due to small sample size), the incidence of heart failure and subclinical cardiotoxicity was lower in the treatment groups (especially in the metoprolol group) than in the control group (31).

Once the diagnosis of chemotherapy-induced cardiomyopathy is established, the oncologist and cardiologist should discuss the patient’s oncologic and cardiac prognosis, while weighing the risks of discontinuing the cardiotoxic agent. The initiation of standard heart failure treatment as well as the discontinuation of the cardiotoxic agent will increase the likelihood of recovery of left ventricular function mainly in cases with type II CRCD. These patients could presumably be treated again with trastuzumab, albeit with close cardiac monitoring (10) 

The guidelines for the treatment of chemotherapy-induced cardiomyopathy are lacking. AHA/ACC guidelines recommend the use of neuro-hormonal antagonists for the treatment of adults with left ventricular dysfunction (32) (Table **2**); however, these therapies were not validated in large randomized studies comprising cancer patients. 

Jensen *et al*. conducted an observational study on 92 patients with advanced breast cancer receiving a cumulative dose of epirubicin amounting to 1000 mg/m². Nine of the patients developed heart failure associated with a significant drop in LVEF and were treated initially with furosemide and digoxin resulting in a transient clinical relief. Eight of these patients were subsequently treated with enalapril or ramipril. After three months of therapy, LVEF increased to normal or near normal levels (33).

In a case controlled retrospective study the use of β-blockers in 8 anthracycline-induced cardiomyopathy was evaluated, and showed similar improvements in left ventricular function (mean LVEF 28% to 41%) compared to 16 matched control idiopathic dilated cardiomyopathy group. Of note, 75 % of the anthracycline-induced cardiomyopathy group received angiotensin converting enzyme inhibitor (ACE-I) (34)

Another study by Tallaj *et al* reported the outcome of 25 patients with anthracycline-induced cardiomyopathy whom treated with standard heart failure therapy. Ten patients received either an ACE-I or ARB and 15 received a combined β-blocker (carvedilol or metoprolol succinate) and ACE-I. The mean survival was 14 years, there were four deaths and one patient underwent heart transplantation. Among the survivors there was a significant improvement in the New York Heart Association (NYHA) class and LVEF (26 ± 9.2% to 35±16.5%, *p *0.022) with a trend towards better improvement in the combination group. (35).

In conclusion, chemotherapy induced cardiomyopathy is a serious complication of cancer therapy rendering the timely identification of high-risk patients the key to reducing this risk. A unified acceptable definition of chemotherapy induced cardiomyopathy adopted by cardiologists and oncologists must be developed. There is also the need for large multicenter trials in order to validate some of the preliminary albeit promising research already conducted in this field. We recommend a close collaboration between the patient’s oncologist and cardiologist to form an individual treatment plan including the standard treatment of heart failure after assessing the oncologic and cardiac prognosis of the patient.

## Figures and Tables

**Table 1.** Chemotherapy Agents Associated with Left Ventricular Dysfunction

Types	Chemotherapy Agents

**Antimetabolites**	Pentostatin
	Cladribine
	
**Microtubule-targeting Drugs**	Cyclophosphamide
**Alkylating agents**	Ifosfamide
	Cisplatin
	
	Mitomycine C
**Antitumor Antibiotics**	Trastuzumab
**Monoclonal Antibodies**	Rituximab(cardiogenic shock)
	Bevacizumab
	Alemtuzumab
	
	Interferon-alfa (prolonged administration)
**Biologic response modifiers**	Interleukin-2 (capillary leak Syndrome)
	Denileukin difitox
	
	Sorafenib
**Multitageted tyrosine kinase inhibitors**	Sunitinib
	Imatininb
	Lapatinib
	Dasatinib
	

**Anthracyclines**	Doxorubicin
	Epirubicin
	Idarubicin

**Table 2.** ACC/AHA Guidelines for the Management of Heart Failure

Stage A	Stage B	Stage C	Stage D
Patients at high risk without structural disease or symptoms of HF	structural heart disease but without symptoms	Structural heart disease with prior or current symptoms	Refractory HF
** Patients examples**			
HTN DM CAD Metabolic syndrome Obesity Familial CM	Previous MI LV remodeling (LVH or low EF) Valvular disease	Shortness of breath Fatigue Reduced exercise tolerance	Marked symptoms at rest despite maximal therapy
** Management**			
Treat HTN, lipid disorders and DM Life style modifications ACEI or ARB in appropriate patients	ACEI or ARB BB Devices in selected patients	Diuretics for fluid retention ACEI BB Aldosterone antagonist ARB Digitalis Hydralazine/nitrates Biventricular pacing Implantable defibrillators	OHT Chronic inotropes Permanent mechanical support End of life care/hospice
